# The histone deacetylase inhibitor panobinostat is a potent antitumor agent in canine diffuse large B-cell lymphoma

**DOI:** 10.18632/oncotarget.25580

**Published:** 2018-06-19

**Authors:** Joana N.R. Dias, Sandra I. Aguiar, Diane M. Pereira, Ana S. André, Lurdes Gano, João D.G. Correia, Belmira Carrapiço, Barbara Rütgen, Rui Malhó, Conceição Peleteiro, João Goncalves, Cecília M.P. Rodrigues, Solange Gil, Luís Tavares, Frederico Aires-da-Silva

**Affiliations:** ^1^ Centro de Investigação Interdisciplinar em Sanidade Animal, Faculdade de Medicina Veterinária, Universidade de Lisboa, Lisboa, Portugal; ^2^ Research Institute for Medicines (iMed.ULisboa), Faculty of Pharmacy, Universidade de Lisboa, Lisboa, Portugal; ^3^ Centro de Ciências e Tecnologias Nucleares, Instituto Superior Técnico, Universidade de Lisboa, Estrada Nacional, Bobadela LRS, Portugal; ^4^ Department of Pathobiology, Clinical Pathology Unit, University of Veterinary Medicine, Vienna, Austria; ^5^ Biosystems and Integrative Sciences Institute, Faculdade de Ciências, Universidade de Lisboa, Lisboa, Portugal

**Keywords:** HDAC inhibitors, panobinostat, LBH589, non-Hodgkin lymphoma, canine lymphoma

## Abstract

Non-Hodgkin lymphoma (NHL) is one of the most common causes of cancer-related death in the United States and Europe. Although the outcome of NHL patients has improved over the last years with current therapies, the rate of mortality is still high. A plethora of new drugs is entering clinical development for NHL treatment; however, the approval of new treatments remains low due in part to the paucity of clinically relevant models for validation. Canine lymphoma shares remarkable similarities with its human counterpart, making the dog an excellent animal model to explore novel therapeutic molecules and approaches.

Histone deacetylase inhibitors (HDACis) have emerged as a powerful new class of anti-cancer drugs for human therapy. To investigate HDACi antitumor properties on canine diffuse large B-cell lymphoma, a panel of seven HDACi compounds (CI-994, panobinostat, SBHA, SAHA, scriptaid, trichostatin A and tubacin) was screened on CLBL-1 canine B-cell lymphoma cell line. Our results demonstrated that all HDACis tested exhibited dose-dependent inhibitory effects on proliferation of CLBL-1 cells, while promoting increased H3 histone acetylation. Amongst all HDACis studied, panobinostat proved to be the most promising compound and was selected for further *in vitro* and *in vivo* evaluation. Panobinostat cytotoxicity was linked to H3 histone and α-tubulin acetylation, and to apoptosis induction. Importantly, panobinostat efficiently inhibited CLBL-1 xenograft tumor growth, and strongly induced acetylation of H3 histone and apoptosis *in vivo*. In conclusion, these results provide new data validating HDACis and, especially, panobinostat as a novel anti-cancer therapy for veterinary applications, while contributing to comparative oncology.

## INTRODUCTION

It has become evident that the importance of small companion animals in the “One Medicine” concept goes beyond their role as reservoirs for infectious diseases and their contribution to human health through the human-companion animal bond. Pet dogs in particular are excellent models for the study of spontaneous degenerative, neoplastic, autoimmune and allergic disorders whose pathophysiology closely resemble their human counterparts [1, 2]. Indeed, when compared with other animal models, the canine model presents unique advantages: diseases are naturally occurring in immune-competent hosts; the size of the animals allows testing therapeutic approaches similar to the ones used in humans; disease mapping and pharmacogenomics are simplified by the organization of dogs into isolated populations with reduced genetic variation (breeds); the relatively fast disease progression rate allows obtaining early conclusions from clinical trials; and the social status of dogs as companion animal allows them to benefit from high quality health care and the ethical exploration of translational approaches [3–5]. Cancer is among the leading causes of death in both dogs and humans. Therefore, efforts towards bringing together veterinary and human medicine for the comparative research of cancer are being pursued [6]. These initiatives are also motivated by the increasing healthcare standards demanded by pet owners, originating the need for novel cancer therapies in veterinary settings [7–9]. One of the most common neoplasias in both species is non-Hodgkin lymphoma (NHL), an heterogeneous group of cancers characterized by the proliferation of malignant lymphocytes [10, 11]. Human NHL is the sixth most common cancer in the United States (U.S), and its incidence nearly doubled since the early 1970 s [11, 12]. Human NHL represents 90% of all lymphomas and 85–90% of cases arise from B lymphocytes. This group of malignancies usually develops in the lymph nodes, but can occur in almost any tissue, ranging from the more indolent follicular lymphoma to the more aggressive diffuse large B-cell (DLBCL) and Burkitt’s lymphoma [13]. Even though current therapeutic options have resulted in improved response rates, the mortality rate is still high [14, 15]. Moreover, the toxicity of conventional chemotherapy often limits its efficacy. Therefore, there has been an increasing interest in the design and development of novel target-specific molecules over the past years [16]. Owing to shared molecular, incidence, genetic, histopathologic and clinical features, canine lymphoma has been proposed as a comparative animal model for the research of novel therapeutic agents and approaches for human NHL [17–20]. Canine lymphoma displays several histological subtypes and patients can manifest a wide range of symptoms. However, most suffer from generalized lymphadenopathy (multicentric form) and are diagnosed with intermediate to high-grade lymphoma, more commonly of B-cell origin. Without treatment, the disease has high mortality [4], requiring prompt chemotherapy to achieve temporary remission and prolonged survival. Yet cure is rarely achieved and the majority of dogs relapse with lethal, drug-resistant lymphoma. Thus, there is an urgent need to develop new treatment strategies in veterinary medicine for refractory disease [21].

Histone deacetylase inhibitors (HDACis) have emerged as an highly efficient new class of anti-cancer drugs [22]. Histone deacetylases (HDACs) catalyze the deacetylation of histones (and other acetyl-lysine-containing proteins), leading to chromatin condensation and transcriptional repression [23]. By inhibiting deacetylating enzymes activity, HDACis regulate aberrant deacetylation and modify gene expression in cancer cells, culminating in cytotoxicity [24–26]. Other putative mechanisms of action include cell cycle arrest, DNA repair inhibition, apoptosis induction and angiogenesis inhibition [27]. Hematological malignancies seem to be particularly sensitive to HDACis [28]. In fact, these agents have shown single-agent activity against T-cell lymphomas, cutaneous T-cell lymphomas, mantle cell lymphomas, and Hodgkin disease [29]. To date, four HDACis have been approved for cancer therapy by the U.S. Food and Drug Administration (FDA) – vorinostat, romidepsin, belinostat and panobinostat [30]. Considering the high efficacy presented by HDACis in targeted human cancer therapy, we conducted the first investigation on their antitumor properties using a canine B-cell lymphoma model. For this purpose, a panel of seven HDACis was initially tested on the well characterized CLBL-1 canine B-cell lymphoma cell line [31, 32] and panobinostat was identified as the most promising compound. Panobinostat was therefore deeply investigated and showed strong *in vitro* and *in vivo* antitumor properties.

## RESULTS

### HDACis suppress cell proliferation and present cytotoxic effects on canine lymphoma

**Figure 1:**
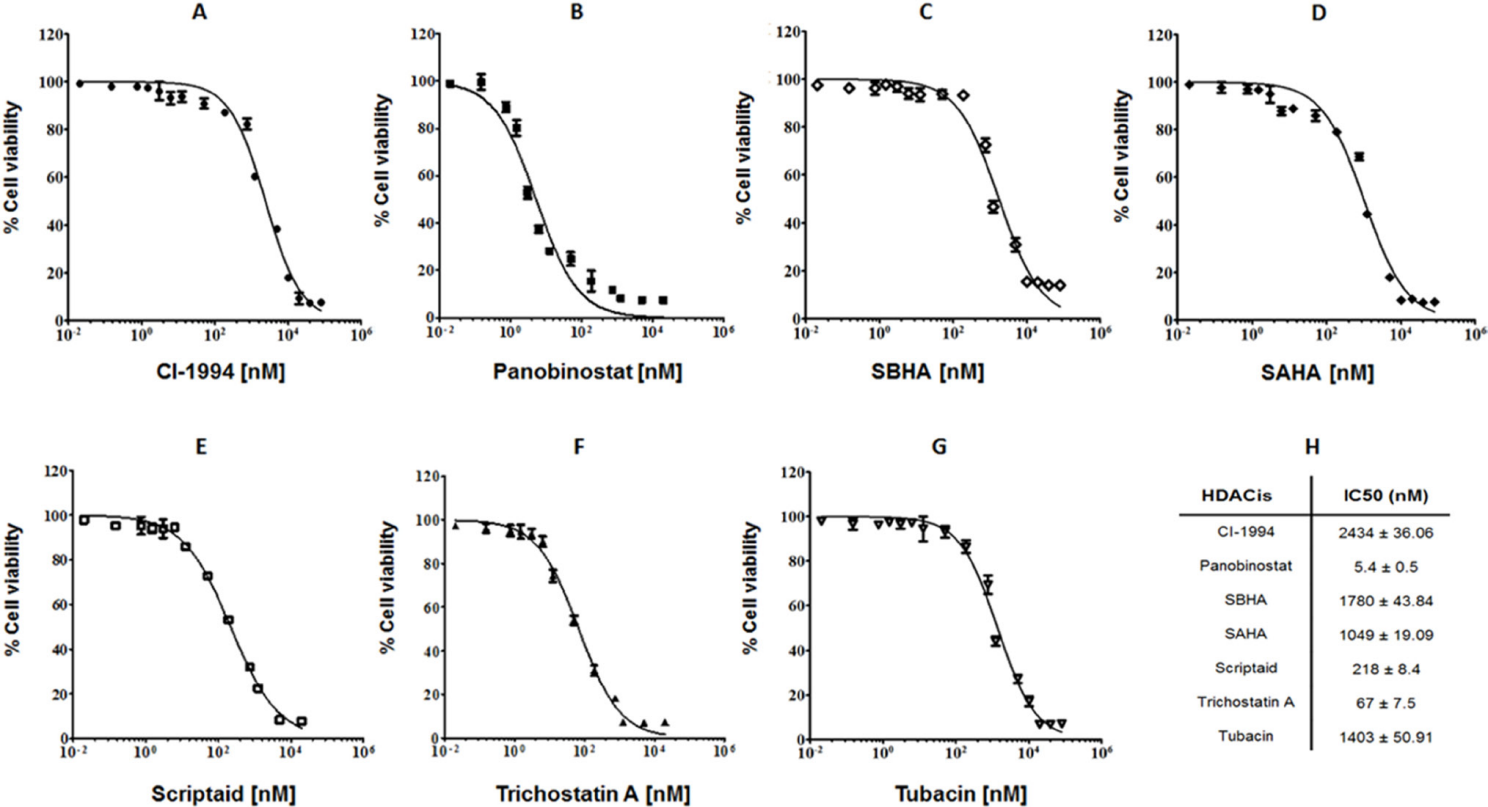
HDACis present cytotoxicity effect on canine B-cell lymphoma CLBL-1 cells (6 × 10^4^) were subjected to the indicated concentrations of HDACis - CI-994, panobinostat, SBHA, SAHA, scriptaid, trichostatin A and tubacin (**A**–**G**). After 24 h treatment, cell viability and proliferation were evaluated with WST-1 reagent. Two replicate wells were used to determinate each data point and three independent experiments were carried out in different days. Best-fit IC50 values of each HDACis were calculated using the log (inhibitor) vs response (variable slope) function (**H**).

Aiming to evaluate the potential cytotoxic effects of HDACis on canine lymphoma we have tested a panel of seven compounds with HDACi activity - CI-994, panobinostat, SBHA, SAHA, scriptaid, trichostatin A and tubacin - in the well-characterized CLBL-1 cell line. CLBL-1 was selected for our study as it is the well-known canine cell line that faithfully represents diffuse large B-cell lymphoma (DLBCL), reproducibly inducing tumors and preserving its phenotype in the xenotransplantation setting [7, 31, 32]. The effect of the tested compounds on cell viability was measured using the WST-1 reagent as described in material and methods section. As shown in Figure 1, all tested HDACi compounds exhibited dose-dependent inhibitory effects on the proliferation of CLBL-1 cells. On the contrary, no evidence of toxicity was detected for vehicle-treated cells. The data obtained clearly demonstrated that panobinostat (IC_50_ = 5.4 ± 0.5 nM), scriptaid (IC_50_ = 218 ± 8.4 nM) and trichostatin A (IC_50_ = 67 ± 7.5 nM) exhibited the higher antiproliferative and cytotoxic activity (Figure 1). The remaining HDACis (CI-994, SBHA, SAHA and tubacin) demonstrated a lower susceptibility to interfere with CLBL-1 proliferation and showed IC_50_ values in the µM range (Figure 1).

### HDACi cytotoxicity is associated with histone acetylation

**Figure 2:**
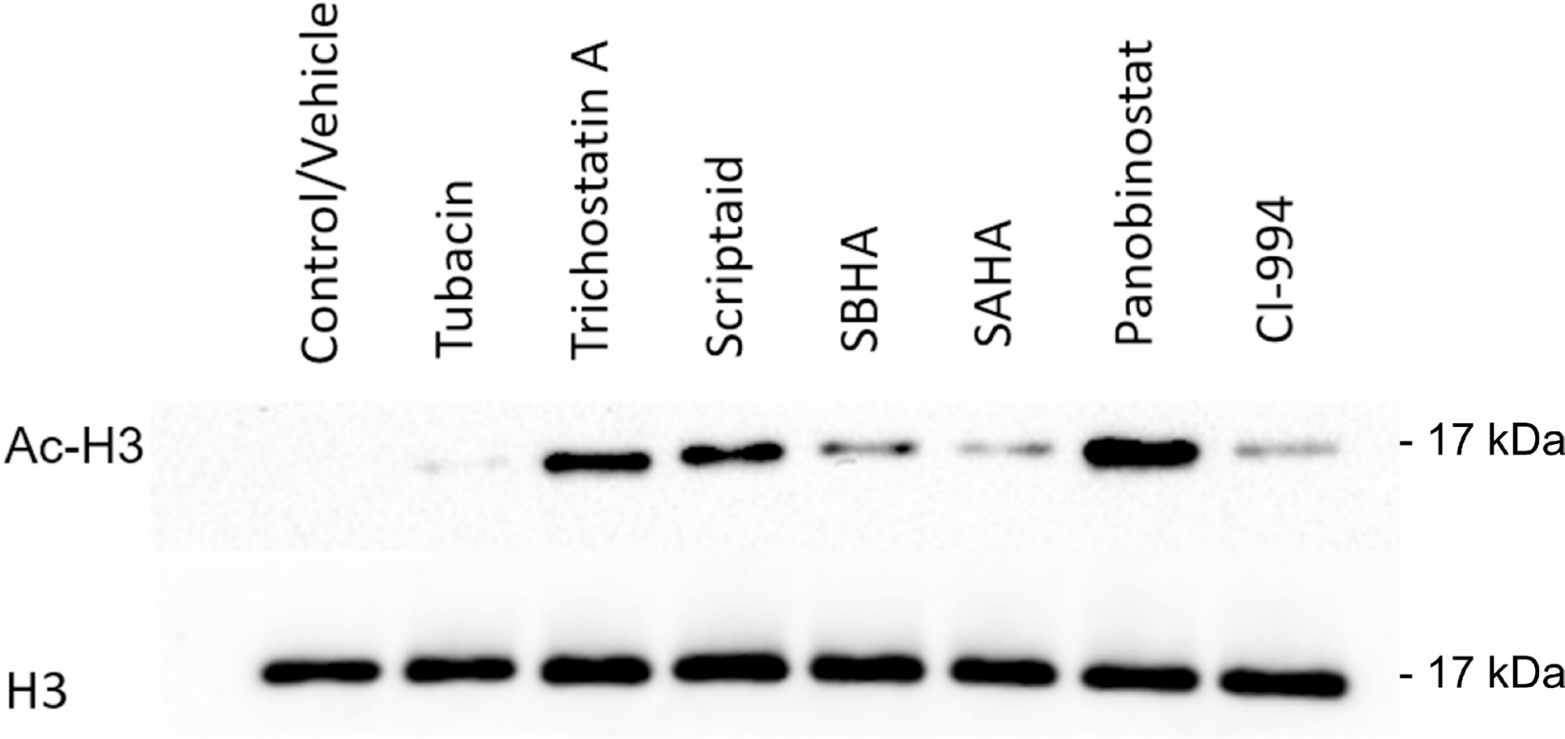
Cytotoxic effect of HDACis correlates with histone acetylation CLBL-1 cells (6 × 10^4^) were exposed to 20 µM of HDACi compounds. After 24 h treatment, cells were harvested for total protein extraction and acetylation of H3 histones were assessed by western blotting with anti-acetyl-histone H3 polyclonal antibody (Ac-H3). DMSO was used as vehicle control and loading was controlled with anti-histone H3 polyclonal antibody (H3). Representative blots are shown.

The primary molecular mechanism of HDACis action is to modify the acetylation status of core histone proteins, leading to chromatin remodeling with consequent alteration in gene expression and cell differentiation. Therefore, to elucidate the mechanism of action of HDACis in the CLBL-1 cell line, we evaluated the acetylation status of H3 histone protein by western blot analysis. As shown in Figure 2, immunoblot analysis demonstrated that CLBL-1 cells presented an hyperacetylation status of the H3 histone protein following 24 h treatment with 20 µM of HDACis, when compared with control vehicle treated cells. Importantly, the H3 histone acetylation levels were consistent with cytotoxic effects of the different HDACis and the compounds that showed the higher potency (Figure 1) promoted the higher effect on acetylation status (Figure 2). Considering the strong anti-proliferative activity and high degree of histone acetylation induction, panobinostat demonstrated to be the most promising therapeutic molecule. To confirm the strong activity in canine B-cell lymphoma, a different cell line, namely 17–71, was also tested with panobinostat. Again, the data obtained (Supplementary Figure 1) demonstrated that panobinostat presents a similar activity profile and histone acetylation induction as shown in CLBL-1. Panobinostat presented therefore a consistent and potent anti-tumor effect against canine DLBCL and was selected for further characterization.

### Panobinostat cytotoxicity is linked to α-tubulin acetylation

**Figure 3:**
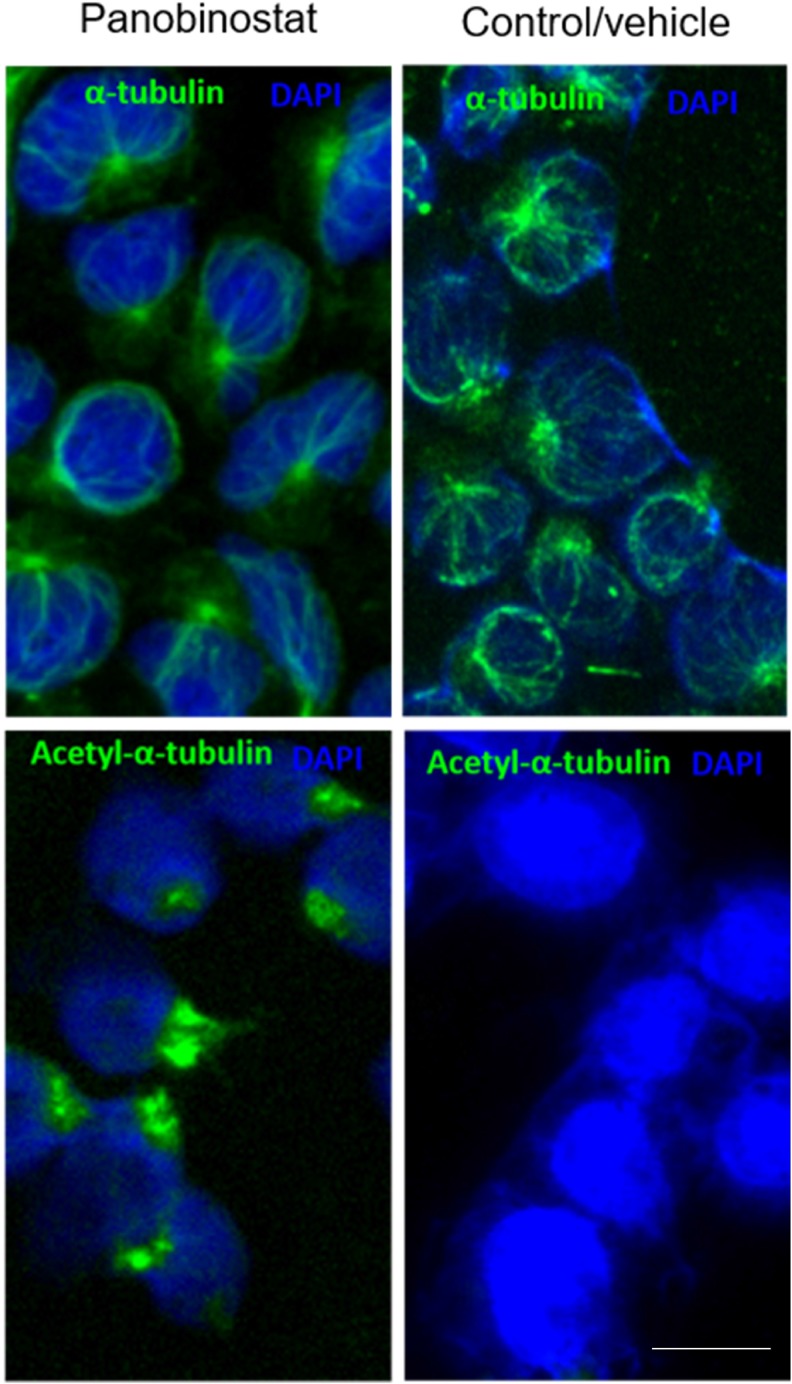
Panobinostat cytotoxicity is linked to α-tubulin acetylation CLBL-1 cells were treated with 10 nM of panobinostat for 24 h and then microtubules were visualized by immunofluorescence labeling using antibodies against tubulin and acetylated tubulin (ac-tubulin) using the appropriate excitation and emission filters as described in the material and methods section. Representative microphotographs with tubulin and ac-tubulin labeling (green) and DAPI stained-nuclei (blue) at 100× magnification are shown. Scale bar, 5 μm.

The activity of HDACis extends beyond chromatin remodeling, and has been associated with the acetylation of non-histone proteins, such as the α-tubulin. The hyperacetylation of α-tubulin results in the stabilization of microtubules and subsequent cytotoxicity [33, 34]. Therefore, to further characterize the mechanism of action of panobinostat, we have assessed the levels of acetylated tubulin (ac-tubulin) in the CLBL-1 cells by immunofluorescence labeling. As shown in Figure 3, immunofluorescence studies revealed an α-tubulin immunolabeling throughout the cytoplasm of both vehicle and panobinostat treated cells, evidencing a normal microtubule network, radiating from the perinuclear microtubule-organizing center. On the contrary, acetylated α-tubulin immunofluorescence studies demonstrated a clear labelling of cells after panobinostat treatment for 24 h, whereas vehicle treated cells exhibited a low basal α-tubulin acetylation (Figure 3). Thus, panobinostat treated cells displayed robust acetylation of α-tubulin with bundling and increased density of acetylated microtubules radiating from the perinuclear region.

### Panobinostat cell death is associated with apoptosis induction

**Figure 4:**
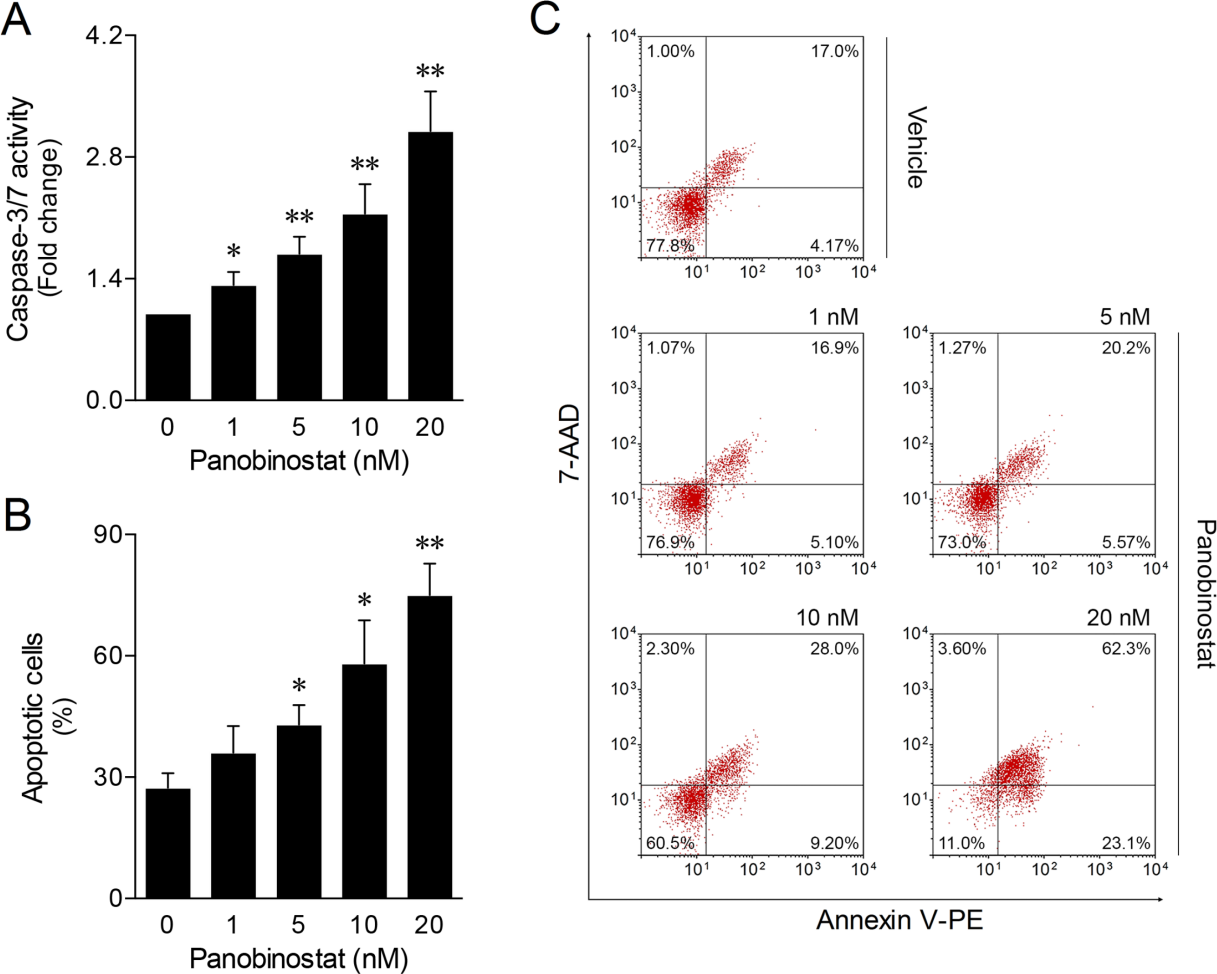
Panobinostat induces apoptosis on canine lymphoma CLBL-1 cells (6 × 10^4^) were subjected to the indicated concentrations of panobinostat. DMSO was used as vehicle control. After 24 h treatment, cells were harvested for apoptosis studies. (**A**) Caspase-3/7 activity was determined using the Caspase-Glo 3/7 assay. Results are expressed as mean ± SEM fold-change to vehicle control cells. (**B**) The percentage of apoptotic cells was determined according to Annexin V/7-AAD (Guava Nexin Assay) staining. Results are expressed as mean ± SEM. (**C**) Representative flow cytometry plots of cells stained for Annexin and 7-AAD are shown. ^*^p < 0.05 and ^**^p < 0.01 from vehicle control cells.

To clarify the nature of CLBL-1 canine lymphoma cell death induced by panobinostat, we measured the levels of caspase-3 and -7 activities and Annexin V/7-AAD staining following 24 h treatment. As shown in Figure 4A, panobinostat treatment induced high levels of caspase activity in a dose-dependent manner. The maximum level of caspase-3/7 activity was seen at 20 nM. Accordingly, a higher percentage of apoptotic cell death was also determined at 20 nM by flow cytometry analysis of Annexin V/7-AAD staining (Figure 4B and 4C). These results are in agreement with the cell viability and proliferation data upon panobinostat treatment, indicating that the cytotoxic activity of panobinostat in the CLBL-1 cell line is consistent with the induction of apoptosis.

### Panobinostat induces tumor regression in a canine lymphoma xenograft mouse model

**Figure 5:**
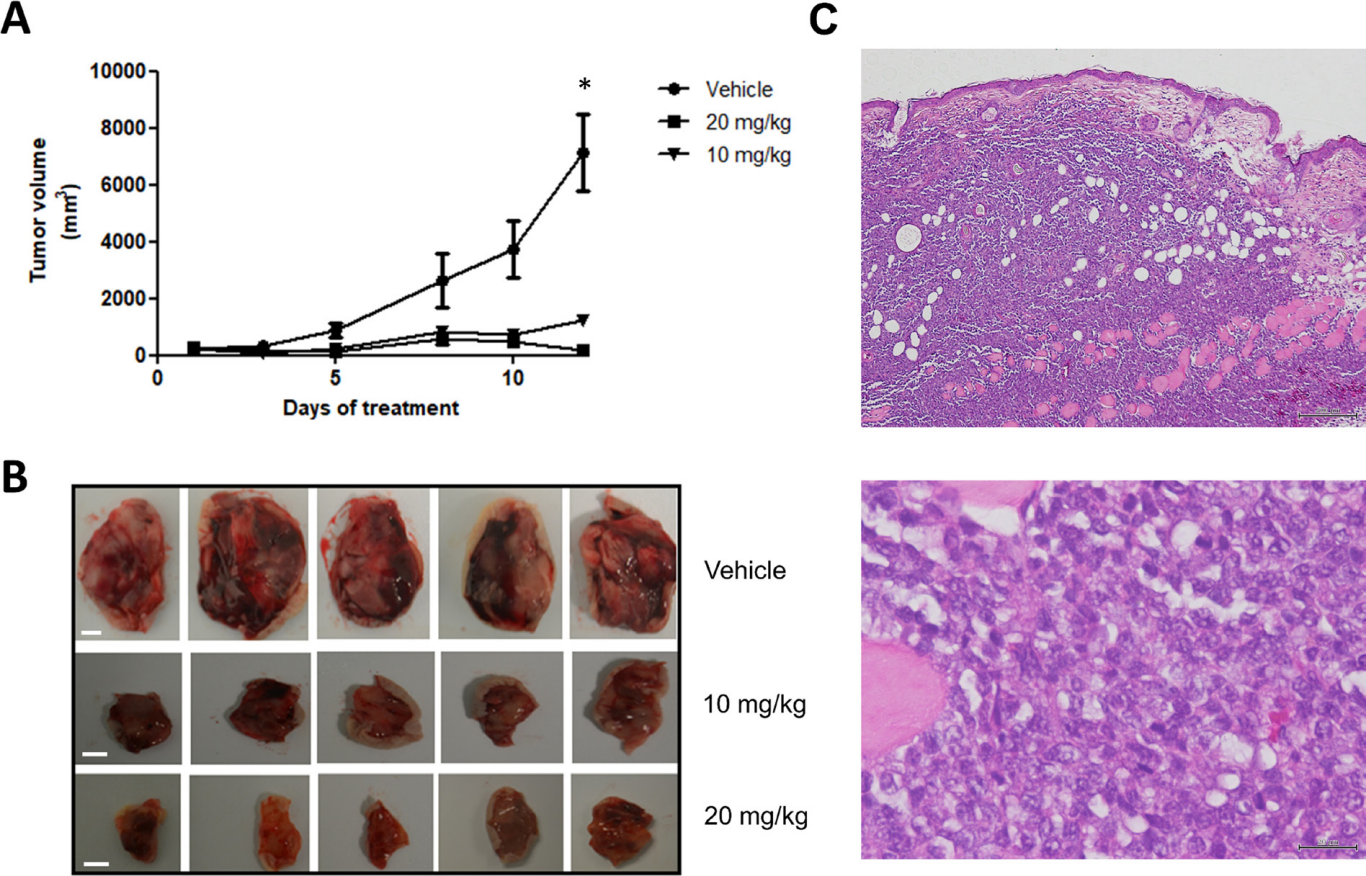
Panobinostat treatment strongly inhibits *in vivo* tumor growth in a canine NHL tumor xenograft murine model SOPF/SHO SCID mice (6-8 weeks-old) were injected subcutaneously with 1 × 10^6^ CLBL-1 cells in a matrigel suspension. When tumors reached ∼100 mm^3^, mice were randomized into three treatment groups: not treated (controls/vehicle only), panobinostat at 10 mg/kg and panobinostat at 20 mg/kg (*n* = 5 per group). Mice were treated with intraperitoneal injections for 2 weeks, 5 days per week. (**A**) Tumor volumes were measured prior to drug administration and after the initiation of therapeutic interventions, three times a week, using a caliper and calculated as (width)^2^ × length (±SEM). The tumor growth curve showed that both treatment groups had a statistically significant tumor growth inhibition compared to vehicle group. ^*^p < 0.05 when compared to the vehicle control treatment. (**B**) Representative images of xenografted tumors were captured at the end of 2 weeks of therapy. Scale bar = 5 mm. (**C**) Representative images of the hematoxylin and eosin (H&E) stained xenograft tumor sections. Upper panel - magnification = 20×, scale bar = 200 µm. Bottom panel - magnification **=** 400×, scale bar = 20 μm.

The antitumor effect of panobinostat on canine B-cell lymphoma was further tested in an *in vivo* murine xenograft model. CLBL-1 suspensions (1 × 10^6^ cells) were injected subcutaneously into the dorsal interscapular region of SOPF/SHO SCID mice. When tumors reached ∼100 mm^3^, mice were randomized into three groups: not treated (controls/vehicle only, *n* = 5), panobinostat at 10 mg/kg (*n* = 5) and panobinostat at 20 mg/kg (*n* = 5) as described in the material and methods section. After two weeks of treatment, panobinostat at 10 mg/kg and 20 mg/kg doses inhibited tumor growth by 82.9% and 97.3%, respectively, when compared to vehicle control treated mice (*p* < 0.05) (Figure 5A and 5B). All xenografts were nodular; infiltration was macroscopically evident, and tumors were highly adherent to surrounding tissues. Microscopically (H&E staining) tumors showed infiltration of the dermis, hypodermis, muscular paniculus and muscle striated by large lymphoid cells of indistinct cytoplasm and nucleus containing several little evident nucleoli (Figure 5C). Moreover, pathologic evaluation described a high degree of necrosis and mitotic activity. These pathological features are characteristic of an high grade lymphoma [31]. No histological alterations were identified in main organs examined (data not shown). Nevertheless, it is important to mention that in the majority of treatment groups, the animals lost an average of 5–10% of their starting body weight, and that some mice from the 20 mg/kg treatment group presented skin dehydration.

### Panobinostat *in vivo* effects on canine lymphoma xenograft tumors

**Figure 6:**
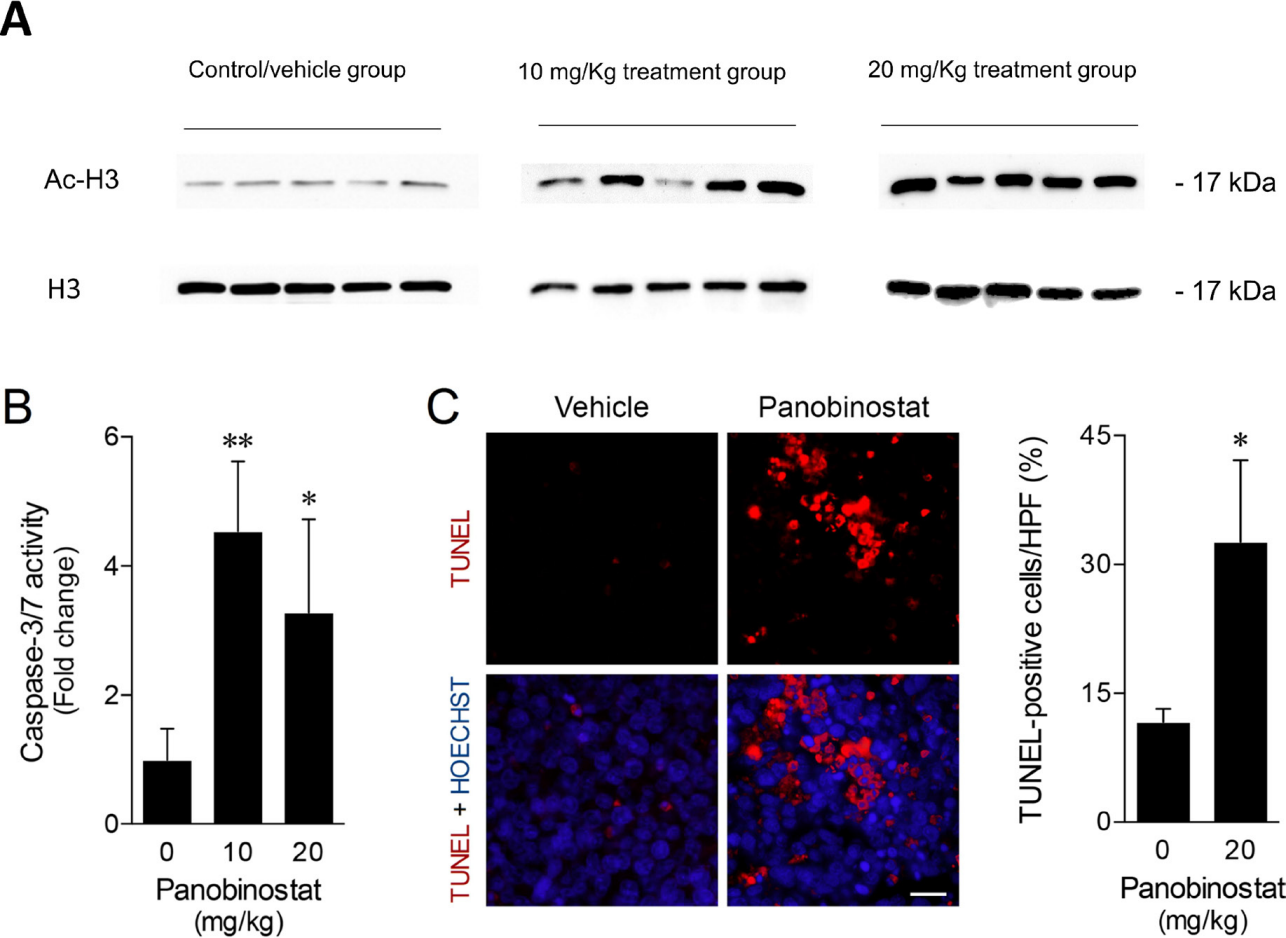
Panobinostat promotes strong *in vivo* anticancer activity (**A**) H3 histone acetylation in total protein extracts from xenograft tumor samples were evaluated by western blot with anti-acetyl-histone H3 polyclonal antibody. Loading was controlled with H3 histone using anti-histone H3 polyclonal antibody. Representative blots are shown. (**B**) Caspase-3/7 activity was evaluated in tumor protein extracts using the Caspase-Glo 3/7 assay. Results are expressed as mean ± SEM fold-change to vehicle treated tumors. (**C**) Apoptosis was evaluated in tumor sections and quantified according to TUNEL-positive cells per high power-field (HPF) (left panel). Representative microphotographs of TUNEL analysis (630× magnification), with apoptotic cells (red) and Hoechst stained-nuclei (blue) are shown (right panel). Scale bar, 15 μm. Results are expressed as mean ± SEM. ^*^p < 0.05 and ^**^p < 0.01 from vehicle control treatment.

To evaluate the panobinostat effects *in vivo*, the acetylation status of the H3 histone protein was evaluated in the tumor xenograft samples by western blot analysis. As shown in Figure 6A, the data obtained demonstrated an increase of H3 histone acetylation on treated tumor samples, compared with control/vehicle treated samples. These differences were strongly evident in 20 mg/kg treated tumor samples. To further characterize panobinostat mechanism of action, apoptosis was evaluated according to caspase activity in tumor protein extracts and TUNEL analysis in histological sections. Consistent with the *in vitro* observations, caspase-3/7 activity was significantly increased up to 4-fold in tumors from treated animals (Figure 6B). Taking that into consideration, TUNEL analysis of *in vivo* tumors was performed after selection of fields without apparent necrosis by the pathologist. Tumor sections from mice receiving panobinostat treatment demonstrated an increase of TUNEL-positive cells as compared to vehicle treated tumors (*p* < 0.05). As shown in Figure 6C, panobinostat treatment at 20 mg/kg was associated with an increase up to 30% of TUNEL-positive cells. Altogether, these results demonstrate that panobinostat exhibits strong antitumor activity in a canine B-cell lymphoma xenograft murine model.

## DISCUSSION

Lymphoma is responsible for significant morbidity and mortality in pet dogs. Its remarkable similarities to human NHL prove clinically realistic opportunities to explore therapeutic protocols that may translate to human clinical trials [3, 4]. To the best of our knowledge, this is the first study exploring HDACis antitumor therapeutic properties for treatment of canine B-cell lymphoma. HDACis have demonstrated anticancer efficacy in both *in vitro* and *in vivo* studies across a range of malignancies, and most impressively in hematological cancers. Consequently, they have undergone a rapid phase of clinical development either as a monotherapy or in combination with other anticancer agents [22, 35]. Indeed, four HDACis have been approved by the FDA for cutaneous T-cell lymphoma and multiple myeloma treatment - vorinostat, romidepsin, belinostat and panobinostat [30]. Regarding the veterinary clinical setting, it has been previously reported by Kisseberth *et al.* the *in vitro* effect of two HDACis, OSU-HDAC42 and SAHA, on a panel of canine cancer cell lines, including canine T-cell lymphoma [36]. In this study, the authors showed that the HDACis induced cytotoxicity, histone acetylation and apoptosis when cells were treated under µM doses [36]. In another study, Wittenburg *et al.* also described that the HDACi valproic acid sensitizes human and canine osteosarcoma cells to the anti-tumor effects of the topoisomerase-II inhibitor doxorubicin. The pharmacokinetics and pharmacodynamics features of this combination treatment was further assessed during a Phase I clinical trial in tumor-bearing dogs, demonstrating the safety and clinical utility of this class of drugs for veterinary oncological applications [37].

Within this context, in the present study we aimed to investigate the HDACis antitumor properties on canine diffuse large B-cell lymphoma. For this purpose, a panel of seven HDACis was initially tested on the well-characterized CLBL-1 canine B-cell lymphoma cell line. The antitumor properties of the tested compounds observed here is in line with previous studies from other authors, both in hematological [37–40] and solid malignancies in other species [38–42]. Indeed, all seven HDACis exhibited dose-dependent inhibitory effects on the proliferation of CLBL-1 cells. Nevertheless, and importantly, with our study we were able to identify three HDACis (panobinostat, trichostatin A and scriptaid), which presented a high cytotoxic activity under the nM range. Moreover, our data demonstrated that the entire panel of HDACis evaluated was able to induce histone H3 acetylation on the CLBL-1 canine lymphoma cell line. Importantly, the levels of acetylated histone status were correlated with the IC_50_ values of the HDACis tested.

Based on the potent cytotoxic activity and induced histone acetylation, panobinostat revealed to be the most promising HDACi against canine B-cell lymphoma (CLBL-1 and 17–71) and was selected for further *in vitro* and *in vivo* studies. Panobinostat is a pan-HDACi, with low nanomolar concentration and high inhibitory activity against all Class I, II and IV HDACs both *in vitro* and *in vivo*, which has undergone extensive preclinical and clinical scrutiny [33]. Panobinostat appears to be at least 10-fold more effective *in vitro* against Class I, II and IV HDACs when compared with vorinostat, the first FDA approved HDACi [43]. A marked anti-tumor activity of panobinostat has been demonstrated across a broad range of cancer cell lines from hematologic malignancies, including cutaneous T cell lymphoma (CTCL), acute myeloid leukemia (AML), chronic myeloid leukemia (CML), multiple myeloma (MM), Hodgkin lymphoma (HL), as well as in solid tumors such as breast, colon, prostate and pancreatic cancer [33, 43]. Importantly, despite its strong antitumor activity at nanomolar concentrations, panobinostat only resulted in apoptosis of normal human cell lines at far greater concentrations, indicating a selective toxicity for cancer cells [44]. In addition, other potential uses for panobinostat include maintenance therapy, its use as a potential radio-sensitizing agent and, combinatory therapy with other immunomodulatory drugs [33].

Noteworthy, the results presented herein have shown that the high efficacy of panobinostat against canine diffuse large B-cell lymphoma at sub-nanomolar concentrations was comparable to what has been described in several human lymphoid malignancies, where IC_50_ doses range from 5.6 to 31.5 nM (mean 11.2 nM) [33, 45].

Several studies also report the involvement of non-histone protein acetylation in a diverse array of cellular processes including protein traffic, apoptosis and cell motility [39, 46]. Therefore, to further investigate HDACi acetylation of non-histone proteins, we evaluated the effects of panobinostat on tubulin acetylation. Our data demonstrated that low doses of panobinostat (5 nM) on CLBL-1 cells led to tubulin acetylation, a marker of microtubule stabilization, and bundle formation.

Apoptosis activation through either the extrinsic or the intrinsic pathway has been shown to be one of the main mechanisms by which HDACis induce tumor cell death [48]. In this context, activation of caspases is a known feature of HDACi-induced apoptosis [47, 48]. In agreement with this, apoptotic cell death of CLBL-1 canine lymphoma cells after panobinostat treatment was strongly evidenced at 20 nM by the activation of caspase-3 and -7, and by the subsequent increase in the percentage of apoptotic cells. These results confirmed that the CLBL-1 canine lymphoma cell line used in this study was a good model to investigate the effects of HDACis and panobinostat. Additionally, it is worth mentioning that the effects of panobinostat observed in this work are attained at concentrations achievable in patient plasma, and are thus highly relevant to the clinical set [39]. Indeed, in a Phase I clinical study on intravenous panobinostat, Cmax reached up to 200 nM [49] and preliminary results [50] from an ongoing Phase I pharmacokinetic study on oral panobinostat, conducted in patients with solid tumors and hematologic malignancies, a steady state Cmax ranged from 15 to 35 nM.

Finally, in order to evaluate the pre-clinical efficacy of panobinostat, we performed *in vivo* studies using a SCID xenograft model implanted with CLBL-1 canine lymphoma cells. Animal data confirmed the antitumor properties of panobinostat, showing that the drug significantly induces apoptosis, while impairing tumor growth. The maximum tumor growth inhibition (TGI) effect was strongly evident at both 10 mg/kg and 20 mg/kg (TGI of 82.9% and 97.3%, respectively). Furthermore, enhanced H3 histone acetylation levels on tumor samples confirmed that *in vivo* tumor growth inhibition was correlated with panobinostat mechanism of action as observed in the *in vitro* studies. It is also important to mention that panobinostat treated groups, namely animals from the 20 mg/Kg treated group, presented mild toxicity signs, similarly to those reported in previously published studies [39]. As stated by Subramanian and collaborators, HDACis are mostly well tolerated, however, due its potent and wide spectrum HDAC inhibition, panobinostat is associated with significant dose-limiting toxicities [51]. No previous clinical studies have been performed in canine patients using panobinostat and other HDACi with similar toxicity profile, such as the FDA-approved vorinostat and romidepsin [52]. For this reason, following preclinical validation of panobinostat, it will be important in the future to carry toxicity studies in dogs using single-agent and CHOP (cyclophosphamide, doxorubicin, vincristine, and prednisone) combination protocols.

In conclusion, the anti-cancer activity of panobinostat demonstrated herein validates HDACis as a novel cancer therapy for canine B-cell lymphoma. Translational and clinical studies will determine the clinical utility and safety of panobinostat as a single/adjuvant agent for the treatment of canine lymphoma. In addition, this work opens up perspectives in comparative oncology as it validates the naturally occurring canine B-cell lymphoma model for translational HDACis research.

## MATERIALS AND METHODS

### Cell lines and reagents

The canine CLBL-1 [31, 32] and 17–71 [53] (kindly provided by Dr. Steven Suter, College of Veterinary Medicine, NC State, Raleigh, North Carolina, USA) B-cell lymphoma cell lines were cultured in Roswell Park Memorial Institute–1640 (RPMI-1640) medium (Gibco, Life Technologies, Paisley, UK) supplemented with 10% heat inactivated fetal calf serum (FCS, Gibco) and penicillin 100 U/ml plus streptomycin 0.1 mg/ml (Gibco). Cell cultures were maintained at 37° C in a humidified atmosphere of 5% CO_2_ (T75-tissue culture flasks, Greiner Bio-One, Kremsmünster, Austria). The Histone Deacetylase (HDAC) Inhibitor Set II, which includes CI-994, Panobinostat (LBH589), SAHA, SBHA, Scriptaid, Trichostatin A and Tubacin, was purchased from Sigma-Aldrich (St. Louis, MO, Cat # EPI009). HDACi stock solutions were prepared at 5 mg/ml, except for CI-994 (10 mg/ml) and SBHA (50 mg/ml) in dimethyl sulfoxide (DMSO) (Sigma-Aldrich) and stored at −20° C. Panobinostat for the *in vivo* studies was purchased from Selleckchem (Houston, TX, Cat # S1030).

### Cytotoxic assay

To determine the effect of HDACis on CLBL-1 and 17–71 cell proliferation, a cell viability assay was performed using the Cell Proliferation Reagent WST-1 (Roche, Basel, Switzerland). Briefly, cells were seeded at a density of 6 × 10^4^ cells/well in 96-well plates in 200 µl of culture medium plus 10% heat-inactivated FCS, and subjected to increasing concentrations (2.5 nM to 20 µM) of the HDAC Inhibitor Set II library or vehicle (DMSO at 0.2% final concentration). After 24 h of treatment, cell viability and proliferation were assessed using the WST-1 reagent, following the manufacturer’s instructions. Absorbance at 450 nm was measured using a plate reader (BMG LABTECH GmbH, Germany). Two replicate wells were used to determinate each data point and three independent experiments were carried out in different days. Best-fit IC50 values were calculated using GraphPad Prism software (version 5.00; San Diego, CA, USA), using the log (inhibitor) vs response (variable slope) function.

### Immunoblotting

After HDACis treatment, cells were harvested, washed twice with PBS and lysed using RIPA lysis buffer (25 mM TrisHCL pH 7.6, 150 mM NaCl, 1% NP-40, 1% sodiumdeoxycholate, 0.1% SDS) supplemented with protease inhibitor cocktail (Roche). Protein extract samples were quantified using the Bradford method (Bio-Rad Protein Assay Dye Reagent Concentrate, Bio-Rad, Hercules, CA, USA) according to the manufacturer’s instructions. Total protein extract samples were separated by 15% SDS-PAGE and transferred to nitrocellulose membranes. After blocking, proteins were probed with the following primary antibodies: anti-acetylhistone H3 (Lys9, Lys14) antibody (polyclonal, rabbit, 1:2500 dilution, Thermo Fisher Scientific, Rockford, IL, USA), anti-histone H3 (polyclonal, rabbit, 1:1000 dilution, Thermo Fisher Scientific), and then with Peroxidase-AffiniPure Anti-Rabbit IgG antibody (polyclonal, goat, 1:10000 dilution, Jackson ImmunoResearch, PA, USA) as a secondary antibody. Proteins were detected by chemiluminescence using Luminata Forte Western HRP (Merck Millipore, Darmstadt, Germany) and acquired using the ChemiDoc XRS+ imaging system (Bio-Rad).

### Evaluation of caspase-3/7 activity

Caspase-3 and 7 activity levels were measured using the Caspase-Glo 3/7 Assay (Promega, Madison, WI, USA). For this purpose, CLBL-1 cells were seeded and treated with 1–20 nM panobinostat as mentioned above. After 24 h of treatment, 100 μL of each cell suspension were transferred into a white 96-well plate, and then mixed with 75 μL of Caspase-Glo 3/7 reagent by orbital shaking for 30 s. Subsequently, the mixture was incubated at room temperature for 30 min, allowing complete cell lysis, stabilization of proluminescent substrate cleavage by caspases, and accumulation of luminescent signal. The resulting luminescence was measured using the GloMax-Multi+ Detection System (Promega). Three independent experiments were carried out in different days.

### Evaluation of apoptotic cell death

The percentage of apoptotic cells after panobinostat treatment was assessed by flow cytometry using the Guava Nexin Assay (Merck Millipore, Darmstadt, Germany), according to manufacturer’s instructions. This assay relies on Annexin V-PE and 7-AAD staining to distinguish between viable (Annexin V/7-AAD double negative), early-apoptotic (Annexin V positive/7-AAD negative), and late-apoptotic/dead cells (Annexin V/7-AAD double positive). Briefly, CLBL-1 cells were seeded and treated with 1–20 nM panobinostat. After 24 h of treatment, cells were collected, centrifuged at 500 g for 5 min, and resuspended in PBS containing 2% FBS. Next, 50 μL of each cell suspension were stained with an equal volume of Guava Nexin reagent for 20 min, at room temperature, protected from light. Sample acquisition and analysis were performed in a Guava easyCyte 5HT flow cytometer using the Nexin software module (Merck Millipore). Three independent experiments were carried out in different days.

### Immunofluorescence microscopy

CLBL-1 cells (6 × 10^4^) were treated with increasing concentrations of panobinostat as described above. After 24 h treatment, cells were fixed in methanol/acetone (1:1) at −20° C for 20 min, washed in PBS, incubated with 0.1% Triton X-100 for 15 min, washed, blocked with BSA 1%/PBS TWEEN 0,2% for 30 min, washed and probed with anti-α-tubulin antibody (clone DM1A, 1:200 dilution, Sigma Aldrich) or anti-Acetyl-Tubulin antibody (clone 6-11B-1, 1:200 dilution, Sigma Aldrich) in PBS for 1 h at room temperature. Then, cells were washed with PBS for 10 min and incubated with anti-mouse IgG alexa 594 secondary antibody (polyclonal, goat, 1:300 dilution, Invitrogen) in PBS for 1 h at room temperature. Nuclear staining was obtained by mounting with DAPI Vectashield (Vector Labs, CA, USA). Cells were visualized using an Olympus IX-50 inverted microscope (Olympus Portugal, Lisbon, Portugal) equipped with Ludl Bio-Point filter wheels, and a 12-bit PCO (Kelheim, Germany) Sensicam QE cool CCD (Ludl Electronic Products, New York, NY, USA). Integrated control of the filter wheel and image acquisition was achieved by Image-Pro Plus 4.5 and Scope- Pro 3.1 (Media Cybernetics, Rockville, MD, USA). Settings for image acquisition (camera exposure time, filters and time interval) were determined by custom-made macros. Images were collected with Olympus 10× or 100× plan objectives (Numerical Aperture = 0.95 and 1.4, respectively).

### Xenograft studies

All animal-handling procedures were performed according to EU recommendations for good practices and animal welfare, and approved by the Animal Care and Ethical Committee of the Veterinary Medicine Faculty. Female 6–8-wk-old SOPF/SHO SCID mice (Charles River Laboratory) were maintained in microisolation cages under pathogen-free conditions. Mice were allowed to acclimatize for at least two weeks prior to the start of the experiment. Suspension of 1 × 10^6^ CLBL-1 cells in PBS with matrigel^®^ (Corning, NY, USA, Cat # 354248) (1:1) were injected subcutaneously into the dorsal interscapular region to induce tumors. When tumors reached a minimum volume of 100 mm^3^, mice were randomly assigned to one of three groups: not treated (vehicle only, *n* = 5), 10 mg/kg panobinostat (*n* = 5) and 20 mg/kg panobinostat (*n* = 5). The tested doses [39, 54, 55] and the vehicle selection (2% DMSO + 48% PEG300 + 2% Tween 80 + ddH_2_O) [56] were based on previously published studies and following manufacturer’s recommendation. Treatment consisted of intraperitoneal injections 5 days per week, over two weeks. Tumor volume and body weight was measured three times per week. Tumor volume was calculated as (width)^2^ × length. Compound activity was determined by tumor growth inhibition (TGI). TGI was determined as the percent change in tumor volume of treated over control animals (%T/C). In the end of the study, tumor samples were finely cut, and either stored at −80° C in RNAlater™ (Invitrogen, Life Technologies, Paisley, UK) or formalin-fixed. Main organs including the liver, kidney, lung, spleen and intestine were collected and formalin-fixed. For immunoblotting and caspases 3/7 detection, RNA later™ (Invitrogen) preserved tumor samples were thawed and processed using tissueLyser II (Qiagen, Hilden, Germany) for tissue disruption, and RIPA lysis buffer supplemented with fresh protease inhibitors (Roche) for total protein extraction. Samples were quantified using Bradford method (Bio-Rad Protein Assay Dye Reagent Concentrate) according to the manufacturer’s instructions, and then evaluation of H3 histone acetylation was carried out as described above. Additionally, tumor protein extracts (15 μg) were used for caspase activity measurement, using the Caspase-Glo 3/7 Assay.

### Histology

Tissues, including tumors, were fixed in 10% buffered formalin were embedded in paraffin, using a Leica tissue processor. Four µm sections were cut from paraffin blocks and stained with H&E. Sections were mounted onto superfrost ultra plus slides (Menzel-Glaser, Braunschweig, DE), for immunohistochemistry.

### TUNEL staining

Apoptotic cells were quantitated in tumor tissue sections, excluding areas of necrosis, using the transferase mediated deoxyuridine triphosphate (dUTP)-digoxigenin nick-end labeling (TUNEL) assay (ApopTag^®^ Red *In Situ* Apoptosis Detection kit; Merk Millipore, Darmstadt, Germany), following the manufacturer’s instructions. Specimens were then counterstained with Hoechst 5 µg/ml, for 10 min, at room temperature. Finally, slides were rinsed and a glass coverslip was mounted using Fluoromount-GTM mounting media (Beckman Coulter Inc., Fullerton, CA). Specimens were examined by fluorescence microscopy using an AxioScope.A1 microscope (Carl Zeiss Microscopy GmbH, Jena, Germany). Images were acquired under 630× magnification, using an AxioCam HRm camera with the ZEN 2012 software (Blue Edition, version 1.1.2.0). Only areas with dense tumor cell mass displaying similar cell density were considered. Quantitation of TUNEL-positive cells was performed using Image J software (http://rsbweb.nih.gov/ij/). Apoptosis frequency was expressed as the number of TUNEL-positive cells per field.

### Statistical analysis

All data are expressed as mean ± standard error of mean (SEM) from at least three independent experiments. For *in vitro* and *in vivo* assays, statistical significances were determined using two-tailed Student’s *t*-test. Values of *p* < 0.05 were considered statistically significant.

## SUPPLEMENTARY MATERIALS FIGURE


