# Supplementary material for: The histone deacetylase inhibitor panobinostat is a potent antitumor agent in canine diffuse large B-cell lymphoma
Source: Oncotarget. 2018 Jun 19;9(47):28586–98. doi: 10.18632/oncotarget.25580 (PMC6033347; doi:10.18632/oncotarget.25580)
Supplement: Supplementary file 1 [file oncotarget-09-28586-s001.pdf]

The histone deacetylase inhibitor panobinostat is a potent antitumor agent in canine diffuse large B-cell lymphoma

SUPPLEMENTARY MATERIALS

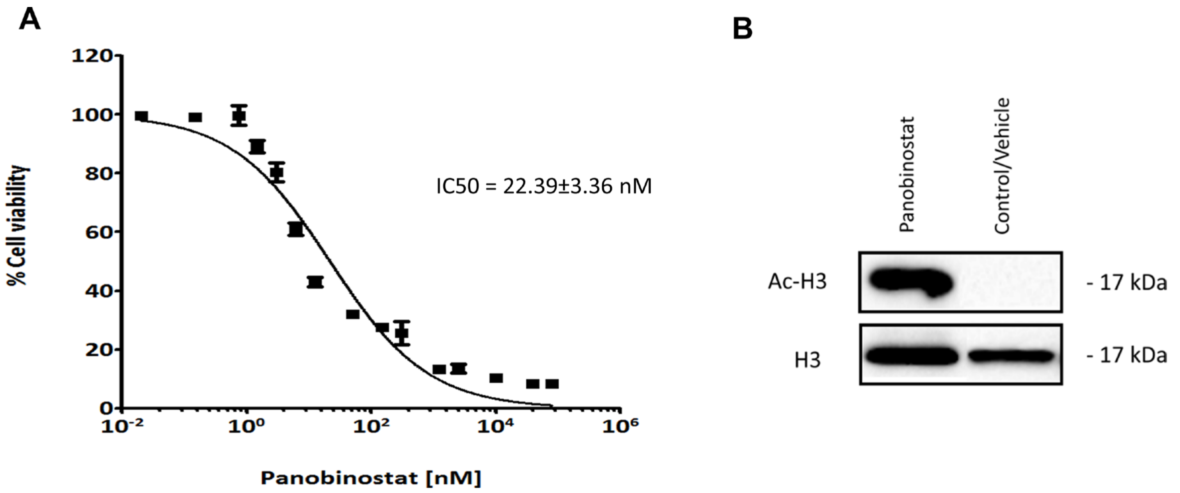

Supplementary Figure 1: Panobinostat presents cytotoxicity on 17–71 canine lymphoma cells and is correlated with histone acetylation. (**A**) 17–71 cells (6×10^4) were subjected to the indicated concentrations of panobinostat. After 24 h treatment, cell viability and proliferation were evaluated with WST-1 reagent. Two replicate wells were used to determinate each data point and three independent experiments were carried out in different days. Best-fit IC50 values were calculated using the log (inhibitor) vs response (variable slope) function . (**B**) 17–71 cells (6×10^4) exposed to 20 µM were harvested for total protein extraction and acetylation of H3 histones were assessed by western blotting with anti-acetyl-histone H3 polyclonal antibody (Ac-H3). DMSO was used as vehicle control and loading was controlled with anti-histone H3 polyclonal antibody (H3). Representative blots are shown.
